# Novel use of the Nathanson liver retractor to prevent postoperative transient liver dysfunction during laparoscopic gastrectomy

**DOI:** 10.1111/ases.12735

**Published:** 2019-08-07

**Authors:** Kazuhiro Hiramatsu, Taro Aoba, Tadahiro Kamiya, Koichi Mohri, Takehito Kato

**Affiliations:** ^1^ Department of General Surgery Toyohashi Municipal Hospital Toyohashi Japan

**Keywords:** gastrectomy, liver, postoperative complications

## Abstract

**Introduction:**

The Nathanson liver retractor (N) has been known to cause postoperative transient liver dysfunction (POTLD) in laparoscopic gastrectomy (LG). To reduce the incidence of POTLD, specifically we added to the retractor the use of a disk (N + D) to reduce the localized pressure, and furthermore repositioned the retractor every 30 minutes (N + D TM) to reduce the liver retraction time. Before and after introducing this retractor, we assessed four consecutive retraction procedures. These included the following disk suspension methods (D), N, N + D, and N + D TM.

**Methods:**

We retrospectively enrolled 85 patients who underwent an LG. In the D, N, N + D, and N + D TM groups, we evaluated the postoperative serum aspartate aminotransferase (AST) and alanine aminotransferase (ALT) values.

**Results:**

For the D and N groups, the AST value significantly increased from the immediate post‐operation time point (IPOT) to the third postoperative day (POD3). Additionally, the ALT value increased from IPOT to POD7. In the N + D group, the only decrease was in the ALT value at IPOT compared to the N group. The N + D TM group decreased in both the AST value from IPOT to POD3 and in the ALT value from IPOT to POD7, compared to the N group.

**Conclusions:**

Our findings demonstrate the importance of reducing both the localized pressure and liver retraction time when using the Nathanson retractor to prevent POTLD during an LG. To make this possible, we successfully introduced the use of both a disk and the repositioning of the retractor at 30 minute intervals.

## 1 INTRODUCTION

Several studies have reported the development of postoperative transient liver dysfunction (POTLD) after laparoscopic gastrectomy (LG).^1^, ^2^, ^3^, ^4^, ^5^, ^6^, ^7^, ^8^ The causes of POTLD after LD have been shown to be numerous, including these two major ones: CO_2_ pressure in the pneumoperitoneum,^5^, ^9^ and the division of an aberrant left hepatic artery (ALHA).^1^ However, various recent reports have suggested another significant cause of POTLD could be simply the effect of direct liver retraction with certain kinds of liver retractors.^2^, ^3^, ^4^, ^6^, ^7^, ^8^, ^10^, ^11^ Furthermore, chronic liver diseases, including severe fatty liver, are considered to increase one's risk with respect to ischemic liver damage.^12^, ^13^

The Nathanson liver retractor (N) has become widely used in LG because of its convenience. However, following the direct compression of the lateral sector of the liver, this retractor appears to often be a cause of POTLD.^10^ We experienced a considerable increase in POTLD as we began using the N following the disk suspension method (D).^6^ Consequently, we changed our approach to prevent liver damage while using it. At first, we attempted to diminish the direct liver compression that resulted from the use of this particular retractor by also utilizing a disk (N + D). This combined approach was based on Shibao et al.'s technique,^7^ with some modification. Next, expecting additional improvement, we periodically repositioned the retractor based on the time which had elapsed (N + D time management [N + D TM]), and not only according to the surgical procedure, as previously reported.^8^

The goal of this study was to observe the change of POTLD in our institution as we systematically switched our methods from D, to N, to N + D and finally to the N + D TM procedures.

## 2 MATERIALS AND METHODS

### 2.1 Patients

Between January 2013 and May 2016, 89 consecutive patients with diagnosis of preoperative clinical T1 (ie, mucosal/submucosal involvement) gastric carcinomas without evident lymph node metastasis, underwent LG at the Toyohashi Municipal Hospital. Of these 89 patients, several were excluded from this study for the following reasons: one was rapidly converted to open surgery, two had their ALHAs sacrificed during surgeries, and one had a severe fatty liver that was diagnosed by preoperative computed tomography.^14^ Therefore, we analyzed a total of 85 patients in the present study.

Informed consent was obtained from all the subjects before enrollment, and throughout this investigation, patient anonymity was fully preserved. The protocol for this research was approved by the Ethics Committee of Toyohashi Municipal Hospital and conformed to the provisions of the Declaration of Helsinki.

### 2.2 Liver retraction procedures

**Figure 1:**
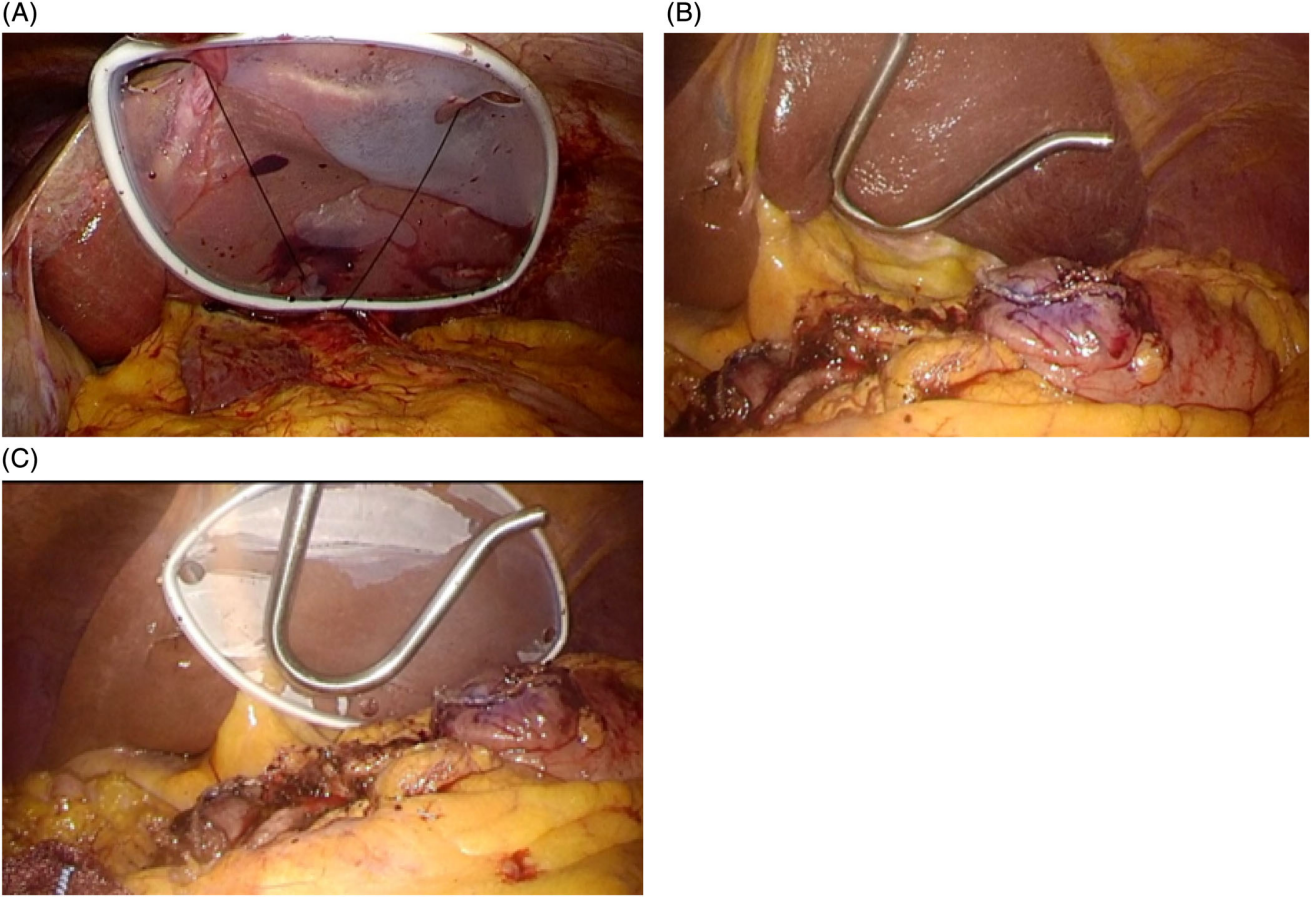
Liver retraction techniques in our institution. A, Liver retraction with a silicon disc fixed between the diaphragmatic crus and the abdominal wall along the costal arch; B, liver retraction with the Nathanson liver retractor; C, liver retraction with the Nathanson liver retractor and the silicon disk

First, for each of the four study groups, the round ligament was lifted and fixed above the adjacent abdominal wall. In the D group, a silicon disk (Hakko, Osaka, Japan) was inserted and fixed between the abdominal wall and the crus of the diaphragm. To this end, we followed the technique of Takemura et al.,^6^ with modification (Figure 1A). More specifically, the disk was a leaf‐shaped device made from a silicon rubber membrane with a flexible shape‐memory frame. In the N group, the retractor was introduced just beneath the xiphoid process and positioned under the liver's lateral sector (Figure 1B). In the N + D group, the retractor and the silicon disk were inserted consecutively. They were both positioned below the lateral sector of the liver. We modified this technique from the procedure reported by Shibao et al.^7^ (Figure 1C). In the N + D TM group, following the positioning of both the retractor and the silicon disk below the lateral sector of the liver, both were subsequently repositioned every 30 minutes. Such changes were done in such a way that liver retraction was never released, and only the retraction point was modified while maintaining the same surgical view.

Two staff surgeons (HK and AT), board‐certified by the Japan Society for Endoscopic Surgery, performed or controlled the LG procedures.

### 2.3 Data collection

Clinical data were retrospectively collected from the medical records and operation videos of each patient. These data included the following 13 pieces of information: (1) age, (2) gender, (3) body mass index (BMI), (4) underlying diseases relevant to hepatic fatty or cirrhotic change (eg, diabetes mellitus, hyperlipidemia, hypertension, and chronic liver disease), (5) operative procedures (eg, distal gastrectomy with B‐I reconstruction, distal gastrectomy with Roux‐en‐Y reconstruction and total gastrectomy), (6) the duration of anesthesia, (7) surgery, (8) pneumoperitoneum, (9) total liver retraction, (10) the amount of intraoperative blood loss, (11) the times required for the completion of the liver retracting device's placement (ie, the time to the disk placement in D group, the time to the N placement in N group, and the time to the N + D placement for the N + D and the N + D TM groups), (12) the serum aspartate aminotransferase (AST) and (13) the alanine aminotransferase (ALT) values before the operation (Pre), and at the immediate post‐operation time point (IPOT), as well as at postoperative days (PODs) 1, 3 and 7.

### 2.4 Study design (changes in the liver retraction procedures during LG in our institution)

**Figure 2:**
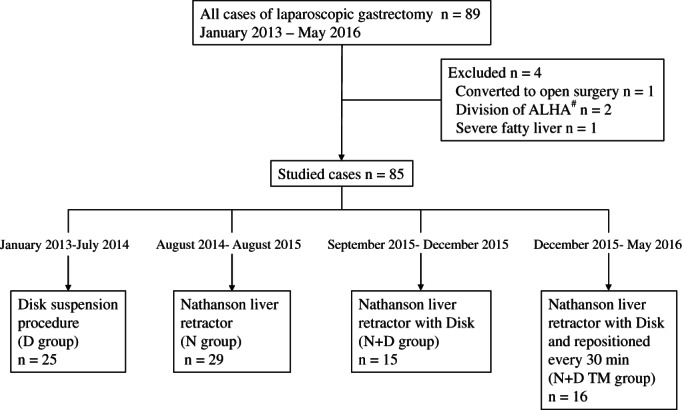
Diagram of patient selection. After four patients were excluded, four consecutive liver retraction procedures were undertaken at our institution between January 2013 and May 2016. After the disk suspension procedure, the Nathanson liver retractor (N) was introduced. Frequent postoperative transient liver dysfunction caused by the Nathanson brought us to make modifications to this technique, followed by two consecutive implemented modifications (ie, N + D and N + D TM group). #ALHA, aberrant left hepatic artery. D, disk suspension procedure; TM, time management

Of our 85 subjects, the 25 in the D group underwent LG between January 2013 and July 2014. Prior to this period, liver retraction was not systematically performed, and therefore the records we used for this study could only start from this period. Subsequently, we introduced the N for 29 cases in our N group between August 2014 and August 2015, and for these cases, we benefited from the N's ease of insertion and flexibility. However, these patients soon exhibited marked POTLD. Therefore, we systematically modified our method of liver retraction for the patients who subsequently underwent LG. The 15 cases undertaken between September and December 2015 were in the N + D group. Finally, an additional improvement was expected upon modification of this procedure for the following 16 cases in the N + D TM group between December 2015 and May 2016 (Figure 2).

For these four different approaches, we performed a comparison of the clinicopathological characteristics and the surgical outcomes that resulted. For each test, two groups were extracted at a time from the four total groups so that comparative tests of each of the six possible pairs of groups could be performed.

### 2.5 Statistical analysis

SPSS ver. 21 (IBM, Armonk, NY, USA) was used to analyze the data. Continuous variables were expressed as the medians and ranges. The χ^2^ test was used to compare the categorical variables between each pair of groups that were extracted from all four studied groups as appropriate. The Mann‐Whitney *U* test was used to compare the continuous variables between each pair of groups. These were extracted from all four studied groups. Results were considered as statistically significant with a *P* < .05.

## 3 RESULTS

### 3.1 Clinical characteristics

Table 1 summarizes the clinical characteristics of the 85 patients. No significant differences were observed between any pair of the four groups in age, proportion of males, BMI or underlying diseases.

**Table 1:** The clinical characteristics of the 85 patients

	D (n = 25)	N (n = 29)	N + D (n = 15)	N + D TM (n = 16)
Age^a^	62 ± 12	65 ± 11	62 ± 14	69.2 ± 8.2
Male gender	13	19	5	10
BMI^a^	22 ± 3.1	23 ± 4.2	23 ± 3.3	22 ± 3.6
Underlying diseases relevant to hepatic fatty or cirrhotic change	7^b^	15	4	5^b^
Diabetes mellitus	3	2	0	0
Hyperlipidemia	1	2	0	1
Hypertension	4	10	3	5
Chronic liver disease	0	1	1	0

a Values are means ± SD.

b Actual number excluding overlap.

Abbreviations: N, Nathanson liver retractor; D, disk suspension procedure; TM, time management.

### 3.2 Surgical characteristics

Table 2 summarizes the surgical characteristics. Each pair of the four groups had comparable operative procedures, and comparable durations of general anesthesia, surgery, pneumoperitoneum, and total liver retraction, as well as similar amounts of intraoperative blood loss. The D group required the longest time for the completion of the liver retraction device's placement (17 ± 7.1 minutes, *P* < .05).

**Table 2:** The surgical characteristics of the 85 patients

	D (n = 25)	N (n = 29)	N + D (n = 15)	N + D TM (n = 16)
Operative procedures				
Distal gastrectomy Bilroth‐I reconstruction	22	20	11	10
Distal gastrectomy Roux‐en‐Y reconstruction	2	3	1	4
Total gastrectomy	1	6	3	2
Duration of general anesthesia (min)^a^	342 ± 66.1	360 ± 55.3	358 ± 66.9	366 ± 84.2
Duration of surgery (min)^a^	280 ± 65.0	306 ± 53.9	304 ± 64.4	308 ± 84.0
Duration of pneumoperitoneum (min)^a^	252 ± 75.3	274 ± 52.9	257 ± 49.7	283 ± 65.1
Duration of total liver retraction (min)^a^	224 ± 71.2	246 ± 52.1	209 ± 49.0	224 ± 64.6
The amount of intraoperative blood loss (g)^a^	67 ± 115.8	86.0 ± 129	38.1 ± 65.5	96.1 ± 180
Time to the completion of the placement of liver lifting device^a^	17 ± 7.1*	4 ± 0.78	3.8 ± 0.97	4.1 ± 0.6

Abbreviations: N, Nathanson liver retractor; D, disk suspension procedure; TM, time management.

a Values are means ± SD.

* *P* < .05 vs all others.

### 3.3 Changes in the postoperative liver functional test results (AST and ALT)

**Figure 3:**
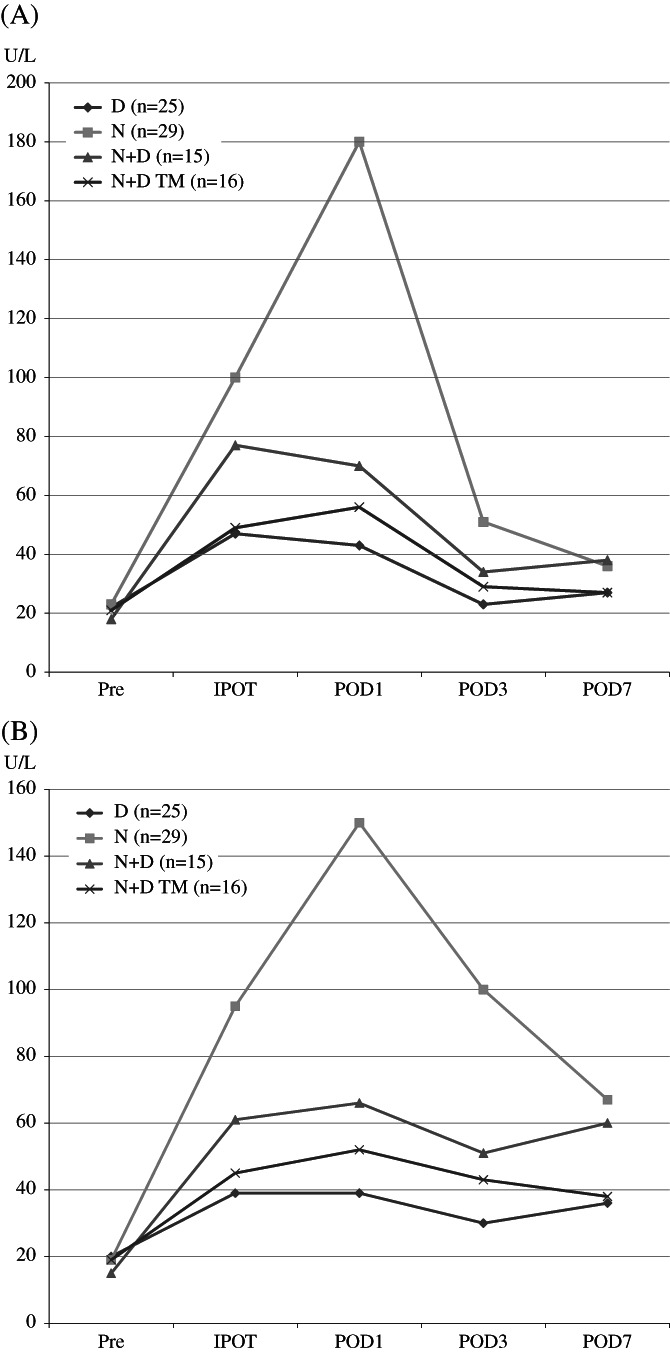
A, Perioperative change of serum aspartate aminotransferase (AST) value in all studied groups. Values are presented as the means U/L. B, Perioperative change of serum alanine aminotransferase (ALT) value in all studied groups. Values are presented as the means U/L. Pre, preoperative day; IPOT, immediate post‐operation time point; D1, postoperative day 1; D3, postoperative D3; D7, postoperative day 7; D, disk suspension procedure; N, Nathanson liver retractor; N + D, Nathanson liver retractor with disk suspension procedure; N + D TM, Nathanson liver retractor with disk suspension procedure and time management

The perioperative changes of the AST and ALT values for all four groups are presented in Figure 3A,B, as well as in Tables 3 and 4, respectively. After the change from the D to the N group, we observed a sharp increase in liver function test values. Specifically, the AST value significantly increased from IPOT to POD3, and the ALT value also increased from IPOT to POD7. However, following the introduction of the N + D procedure we observed a decrease in the liver function test values compared to that of the N group, although this difference was only significant for the decrease in the ALT value on POD3. We also observed that the N + D TM procedure significantly decreased liver function test values. Such findings were evident in both the AST value from IPOT to POD3 and in the ALT value from IPOT to POD7, compared to those of the N group. Direct comparisons between the N + D and N + D TM groups demonstrated that the latter exhibited a decreased AST value at IPOT and a lower ALT value on POD3. Neither the N + D nor the N + D TM groups showed any differences compared to the D group regarding postoperative AST values. In addition, only the N + D group exhibited significant increases in ALT values at IPOT, compared to the D group.

**Table 3:** Perioperative change of AST value and comparative test between each pair

		Pre	IPOT	D1	D3	D7
AST (U/L)^a^	D	22 ± 5.2	47 ± 14.8	43 ± 14.6	23 ± 15.5	27 ± 11.2
	N	21 ± 6.4	103 ± 96	176 ± 17 × 10	51 ± 41	36 ± 22
	N + D	18 ± 3.6	84 ± 61	78 ± 58	37 ± 31	38 ± 26
	N + D TM	21 ± 4.0	45 ± 16	50 ± 25	29 ± 21	27 ± 8.3
*P* values	D vs N	.269	.00101*	3.11 × 10^‐5*^	.000644*	.44
	D vs N + D	.0687	.0692	.162	.093	.229
	D vs N + D TM	.717	.64	.831	.295	.883
	N vs N + D	.232	.757	.083	.4	.594
	N vs N + D TM	.377	.0025*	.000429*	.0177*	.355
	N + D vs N + D TM	.0705	.0397*	.143	.463	.227

Abbreviations: AST, aspartate aminotransferase; D, disk suspension procedure; D1, postoperative day 1; D3, postoperative D3; D7, postoperative day 7; IPOT, Immediate post‐operation time point; N + D Nathanson liver retractor with disk; N + D TM, Nathanson liver retractor with disk and time management; N, Nathanson liver retractor; Pre, Preoperative day.

a Values were expressed as mean ± S.D.

* *P* < .05.

**Table 4:** Perioperative change of ALT value and comparative test between each pair

	Group	Pre	IPOT	D1	D3	D7
ALT (U/L)^a^	D	20 ± 8.9	39 ± 18.0	39 ± 19.8	30 ± 24.8	36 ± 19.6
	N	17 ± 6.8	95 ± 77	153 ± 15 × 10	104 ± 98	67 ± 43
	N + D	15 ± 4.9	69 ± 52	75 ± 60	54 ± 58	60 ± 52
	N + D TM	19 ± 6.1	43 ± 23	47 ± 30	38 ± 31	38 ± 22
*P* values	D vs N	.394	.000242*	9.78 × 10^−5^ *	9.12 × 10^−5^ *	0.00364*
	D vs N + D	.145	.0456*	.0648	.061	.105
	D vs N + D TM	.968	.989	.894	.947	.649
	N vs N + D	.51	.45	.151	.000151*	.785
	N vs N + D TM	.348	.00395*	.00148*	.000152*	.00737*
	N + D vs N + D TM	.0623	.0889	.0659	.000153*	.114

Abbreviations: ALT, alanine aminotransferase; D, disk suspension procedure; D1, postoperative day 1; D3, postoperative D3; D7, postoperative day 7; IPOT, Immediate post‐operation time point; N + D Nathanson liver retractor with disk; N + D TM, Nathanson liver retractor with disk and time management; N, Nathanson liver retractor; Pre, Preoperative day.

a Values were expressed as mean ± S.D.

* *P* < .05.

## 4 DISCUSSION

Use of the N has rapidly and widely spread due to its ease of insertion and considerable variability in the retraction direction. Such features are not available with other devices. However, it has been shown that the use of this particular device is a significant culprit in causing POTLD.^4^ Moreover, there have been reports linking the N to a few cases of severe postoperative liver dysfunction.^10^, ^11^ Clearly, while this device continues to be used, POTLD should be prevented as much as possible.

It has been shown that two factors cause POTLD in LG. First is the magnitude of direct and narrowly focused compression affecting the surface of the liver due to the liver retraction.^4^ Second is the duration of the liver retraction.^8^ The former mechanism is caused by the linear compression of the liver surface with the Nathanson's unique columnar metal. Liver retraction can also be performed with other devices, such as Penrose drains,^4^ and silicon disks.^6^, ^7^ With these devices, the contact sites on the liver do not form a straight line but a flat surface. These devices considerably disperse the hepatic surface pressure compared to the N. Therefore, liver ischemia is believed to occur less frequently with these devices. Similarly, our study demonstrated that the increases in the liver enzymes of the D group were less than those in the N group. However, unlike the N, most of these devices are required to be fixed within the body at the time of insertion.^4^, ^6^, ^7^ Consequently, additional time is needed, and it is difficult for these devices to change the surgical field of view during an operation compared to the N. In our study, the fixation time required for the device in the D group was significantly longer than other groups. We note that Shibao et al.^7^ demonstrated a successful approach to liver damage reduction by interposing a silicon disk on the liver where it interfaces with the retractor. Such a technique would solve the problem of the highly focused localized pressure that results from the use of a snake‐type retractor similar to the Nathanson retractor.^7^ In our institution, we adopted and modified this method, achieving favorable results in the N + D group of this study. While the average AST and ALT values were decreased compared to that of the N group, the differences were not statistically significant. However, we nonetheless felt that further modifications would be worthwhile to achieve a stable prevention of POTLD.

Therefore, we developed a second mechanism for preventing POTLD, namely modifying the duration of liver retraction, when using any liver retraction device.^8^ We regard this as especially important for devices that exert highly localized pressure, such as the N. Kitajima et al.^8^ reported that frequent repositioning of the N as the surgical procedure progressed is considered necessary, to reduce the retracting time. However, with this procedure, retraction time can be quite variable and there is a risk of prolongation in case of an increase in the entire operation time. Therefore, a system which allows more consistent and regular retraction time control may be indispensable. Our 30 minutes intermittent retraction protocol could represent one of the possible solutions to this challenge.

Another important measure that can help reduce the incidence of POTLD is for surgeons to more fully pay attention to changes in the color of the liver's surface. Any such discoloration should motivate a release in retraction. Unfortunately, it has been shown that POTLD has already progressed to a certain degree by the time the liver's color turns purplish.^8^ Hence, there is clearly a need for a method of knowing when to interrupt liver retraction, before such a change of color can be observed, and the intermittent repositioning of 30 minutes we are suggesting in this paper can be one of the solutions.

In Japan, the Pringle methods currently used^15^ involve intermittent clamping of the porta hepatis for 15 minutes during hepatectomies.^12^ However, changing the operative field of view every 15 minutes may encumber the progress of the surgery. Although 15 minutes is generally considered to be the longest allowable time in animal experiments,^16^ it has recently been shown that a 30 minutes clamp time is not a problem compared to a 15 minutes alternative in human randomized controlled trials (RCTs).^17^ Therefore, we adopted a 30 minutes interval for intermittent repositioning. Eventually, the N + D TM group experienced no prolongation of the operation time compared to that of the N + D group.

Our study does have several limitations. First, the data collection for every subject was retrospective, and the operations that were involved using the different protocols may have been affected by a learning curve among the participating surgeons. However, the risk of bias from this aspect is considered to be minimal as no differences were observed across the many procedures involved regarding operation time, liver retraction time or the amount of bleeding. Second, due to the modest number of subjects involved, this study could be considered somewhat statistically underpowered, primarily because the conclusions presented here are based on the evidence from small numbers of subjects in each of our four subgroups. Specifically, these numbers ranged between 16 and 29 cases. Finally, setting the duration between each repositioning at 30 minutes is not known by any rigorous methods to be an optimal choice. The present study represents to date the only report on this subject. Therefore, additional studies would be highly productive. For example, RCTs are needed to determine whether the 30 minutes duration is optimal.

In conclusion, we developed a simple measure to help prevent the Nathanson retractor from causing POTLD during LG. To this end, we found that it is essential to reduce both the localized pressure and the liver retraction time. To accomplish these goals, in the present study, we introduced both a disk and an intermittent repositioning protocol of the retractor every 30 minutes.

## CONFLICT OF INTEREST

Dr. Kazuhiro Hiramatsu, Dr. Taro Aoba, Dr. Tadahiro Kamiya, Dr. Koichi Mohri and Dr. Takehito Kato have no conflicts of interest or financial ties to disclose. All authors made a significant contribution to this report. We have no funding or grant to declare for this paper.

## AUTHOR CONTRIBUTIONS

Kazuhiro Hiramatsu designed the study, and wrote the initial draft of the manuscript. Taro Aoba contributed to analysis and interpretation of data, and assisted in the preparation of the manuscript. All other authors have contributed to data collection and interpretation, and critically reviewed the manuscript. All authors approved the final version of the manuscript, and agree to be accountable for all aspects of the work in ensuring that questions related to the accuracy or integrity of any part of the work are appropriately investigated and resolved.
